# Author Correction: Eruption of ultralow-viscosity basanite magma at Cumbre Vieja, La Palma, Canary Islands

**DOI:** 10.1038/s41467-022-31649-x

**Published:** 2022-07-07

**Authors:** Jonathan M. Castro, Yves Feisel

**Affiliations:** Institute of Geosciences, University of Mainz, Becherweg 21, Mainz, D-55099 Germany

**Keywords:** Volcanology, Geochemistry

Correction to: *Nature Communications* 10.1038/s41467-022-30905-4, published online 8 June 2022.

The original version of this Article contained an error in the Acknowledgement Section, which incorrectly read

‘The authors thank N. Perez and M. Pankhurst for a sample permit’.

The correct version removes this sentence. This has been corrected in both the PDF and HTML versions of the Article.

