# Supplementary material for: Eruption of ultralow-viscosity basanite magma at Cumbre Vieja, La Palma, Canary Islands
Source: Nat Commun. 2022 Jun 8;13:3174. doi: 10.1038/s41467-022-30905-4 (PMC9177865; doi:10.1038/s41467-022-30905-4)
Supplement: Supplementary file 2 — Supplementary Information [file 41467_2022_30905_MOESM2_ESM.pdf]

Supplementary Information

This document provides supplementary information in support of the results and discussion in "Eruption of ultralow-viscosity of basanite magma at Cumbre Vieja, La Palma, Canary Islands". The material consists of Supplementary Figures depicting the results of viscosity measurements in an Arrhenian plot, textural methods, field lava observations, and an Extended Methods section that thoroughly details applied geochemical, textural, videography, and geothermobarometric methods of this work.

Supplementary Figure 1. Rheological data (solid squares and circles) on the 2021 Cumbre Vieja's effective viscosity cast as an Arrhenius plot. The fit-line is based on the high-temperature data (symbols) and reflects a natural logarithmic relation indicated by the equation at the upper left. The correlation coefficient R^2 of 0.98 indicates a strong Arrhenian character to the viscosity versus temperature relation. Solid red circles are crystal-rich melts that were not included in this data analysis.

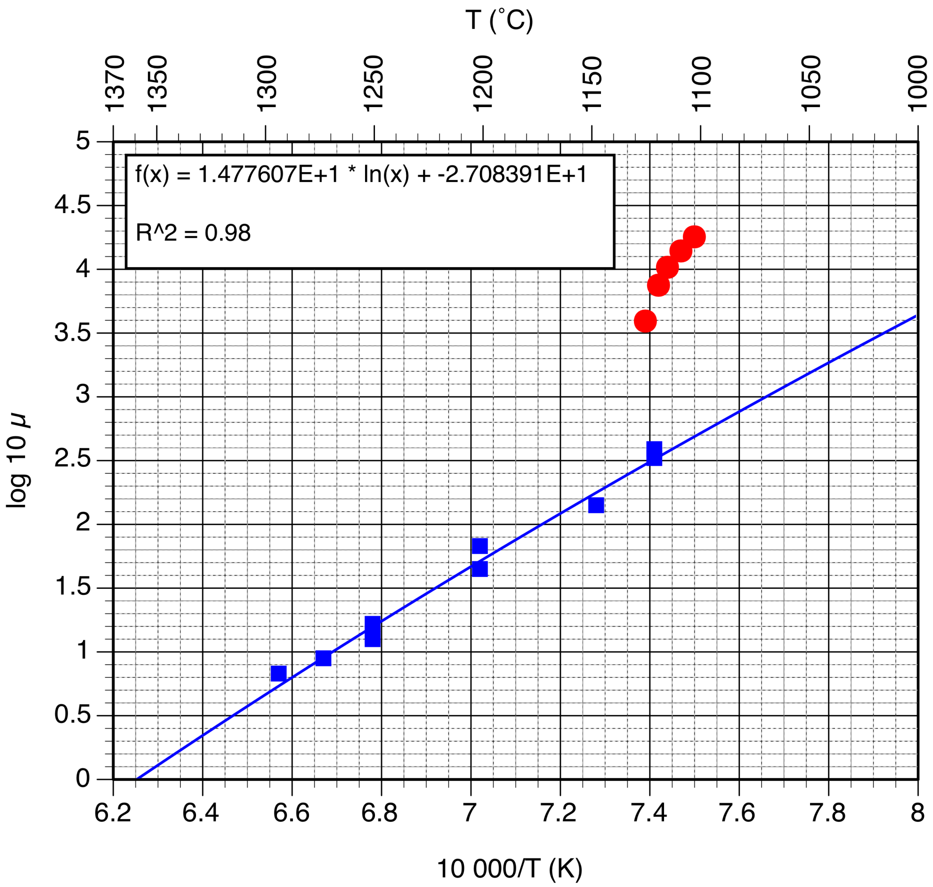

Extended Methods

Samples and Rheology

The samples investigated comprise fine to coarse ash from air fall tephra deposits sampled in both proximal (~3 – 4 km distant from vent) and distal (Santa Cruz de la Palma:~12 km distant) locations with respect to the 2021 El Paso (La Palma, Cumbre Vieja) vent. These materials were collected during the week of 14-20 November 2021 from flat surfaces that include the field vehicle and cardboard sheets set out to capture the ash as it fell. That there was no rain during this week, the samples have not undergone any post deposition alteration by surface processes. Samples were swept into airtight plastic bags shortly after deposition and later transferred to glass jars.

Rheological experiments were performed to determine the high-T, crystal-free and crystal-bearing viscosities using a Brookfield DV3T rotating cylinder rheometer coupled to a Thermconcept vertical elevator tube furnace. A typical experimental run involved filling tephra into a Pt crucible (350 ml) and melting it at 1200˚C, while the spindle remained suspended outside and above the crucible. Temperatures were monitored with two thermocouples (TC), one internal to the furnace and an external K-type chromel-alumel thermocouple that was introduced into the furnace through a port in the furnace's roof. The external TC was lowered to both the surface of the melt and inserted to the melt base via a platinum sleeve to document any potential gradients across the sample. Each TC measured with good precision ($\pm$ 3˚C) however an offset was observed amounting to 50-60˚C between the external TC (reading higher) and the furnace TC. We attribute this T-offset to a vertical thermal gradient within the furnace arising from the opening at the top of the furnace where the spindle is introduced into the furnace. This temperature difference persisted across the entire range investigated and thus all reported temperatures in this work are the external TC temperature. Melting and eventual filling of the crucible with said melt proceeded in steps, comprising repeated additions of ash (in amounts of ~50-70 g each) to the crucible via an opening in the bottom of the furnace. Once the molten tephra reached a level to within 4 mm of the surface of the crucible, we introduced the spindle by lowering it with a gear-drive built into the frame of the rheometer. Care was taken to ensure that the insertion depth was the same for all measurements (ca. 2 mm above the base of the crucible). Viscosity measurements were performed at different temperatures (1110˚C-1300˚C) for durations typically on the order of several hours, until the viscosity reading reached a plateau which was interpreted to reflect that the melt attained its crystal-melt equilibrium at the corresponding temperature. Two cylindrical spindle end pieces were used to perform concentric cylinder viscometry, each being of made of Pt-Rh (800/200 alloy) for its temperature stability and chemical inertness, yet having different diameter (3 mm vs. 9 mm) to expand the range of possible melt viscosities for given rheometer settings.

The Brookfield DV3T measures viscosity on the principle of Couette flow by measuring the torque exerted on the concentric spindle as it rotates in a bath of melt and then converting that torque value to viscosity. The rheometer's torque

reading reflects the shear force arising from the contact of the melt with the surface of the spindle for various rotation rates—cast as revolutions per minute—and thus, the greater the surface area in contact with the melt achieved by using larger diameter spindles, the lower the viscosity that can be measured for a given optimized rotation rate and associated torque. We used the thin 3-mm spindle to investigate viscosities in the range of approximately 1000 to 100000 Pa s, and the larger spindle to measure viscosities ranging from less than 10 to about 100 Pa s.

The instrumental error on viscosity measurements is a function of the torque measurement, and is minimized for torque values in the range of 10-90%. Thus, for all measurements we aimed to use a spindle size and rotation rate for a given temperature interval to achieve sufficient torque on the sample/spindle arrangement; most measurements fell in the range of 10-20% torque. The rheometer also provides an instrument error range, or measurement precision, that is typically less than ±10 to about 230 Pa s. Thus we consider the higher end of this range to be the maximum error on our measurements. The accuracy of viscosity estimates was determined by calibration with a range of viscosity standard fluids (Cannon N270000, N1900000, N2400000) whose individual temperature-dependent viscosities were determined in the factory and span the expected and permissible range of natural basaltic lava viscosities[1]. Calibrations were made for each spindle end piece by submersing the spindle in the viscosity fluid and monitoring both the fluid temperature (15˚C-30˚C) and torque during the measurement. Factory supplied fluid viscosities were reproduced at the relevant temperatures to within $\leq$ 10% of the values on the standard fluid data sheets. Calibration measurements were repeated 3-5 times before unknown measurements were made and then again, after high-T natural experiments to check for instrument drift. We observed no significant differences in the viscosity values made before and after unknown natural melt measurements. Thus, instrument drift was null, and all high temperature measurements on natural melts are very well constrained in terms of their absolute viscosities.

Melt-crystallinity determinations

Given the temperature range investigated and the attendant physical changes that the melt undergoes across this thermal window, crystals likely nucleated and grew at some point during the experiments. Thus, depending on whether the melts were pure or if they contained solids, the measurements could reflect either pure-melt (liquid only) and relative or bulk viscosities that mark a suspension rheology. The most profound effect of growing crystallinity is the step-function like change in viscosity observed at about 1125˚C and continuing to 1110˚C (Fig. 6 in main body text). This sharp viscosity increase is well documented in other systems reported in the literature[5] and reflects the development of high suspension viscosity and yield stress in the suspension due to strong crystal-crystal interactions, which in turn result in high suspension shear stress. Added effects of high crystallinity include the onset of non-linear rheology (e.g., shear-thinning behavior), which again manifests interactions between crystals and melt and potential shear heating effects. It was

therefore important to establish whether and when the crystal content in the rheological experiments reached a level to where such effects would strongly influence viscosity measurements (~40% by volume)[2] and accordingly refine our interpretation of viscosity data (e.g., being relevant to early magmatic processes like rise and emergence versus, long-lived flow emplacement).

Melts at the highest of experimental temperatures (1300-1200˚C), were not subject to crystallization and thus rheological measurements record pure-melt viscosities. This was confirmed by pulling the spindle from the melt residing at 1200 °C and quenching the adhered melt in water. Subsequent microscopic evaluation of these glasses revealed only a small fraction (<<1 vol.%) of small (<50 μm diameter) sparse anhedral oxide minerals that we interpret to have been undergoing dissolution at the time they were quenched in the glass. However, at temperatures below 1200˚C, we observed crystallinities that varied systematically with temperature.

In order to constrain the crystallinities of experiments as a function of temperature, and to assess the potential related rheological influence of those crystals on viscosity measurements, we performed a separate series of crystallization experiments in the range of 1265˚C to 1123˚C. Crystallinity experiments were performed by loading small quantities (0.09-0.1 g) of the starting basanite ash into five, 5-mm diameter Pt cups and then placing these in a room-temperature Nabertherm muffle furnace. The temperature was monitored with both an internal TC and external K-type TC, which was positioned directly next to the Pt cups (~4 cm) and the offset between the two TCs was about 13˚C. With the crucibles in place, the furnace was then heated to 1265 ˚C over one hour and let dwell at this temperature for fifteen minutes. This resulted in melting. At the end of this first dwell period, we removed one crucible with Pt-tongs and quenched it in a water bath. The furnace temperature was then brought to a series of lower temperature set points and let dwell, each time resulting in the removal of one crucible. The temperature steps were made a close as possible to those of the rheological experiments: 1213˚C, 1163˚C, 1138˚C and 1123˚C and changes between these were instigated automatically over a duration of no more than five minutes by the furnace's temperature control unit. With the exception of the last two temperature steps (1138˚C and 1123˚C), the dwell period at each step was always fifteen minutes. However, we chose to employ 30 minute dwells to the lowest two temperatures to enable the melt to reach a steady state crystallinity. Because of the basanite's low melt viscosity and commensurate high rates of chemical diffusivity, we expected kinetic limitations to crystal growth to be negligible. However circumstantial evidence suggests that despite this long (~30 min) dwell, that crystallinity did not reach its true maximum due to time; this was evidenced by a continually rising effective viscosity in rheology experiments at this temperature, the duration of these experiments lasting hours (Fig. 6). Inspection of melts extracted at different temperatures does however establish that all melts investigated were bubble free, meaning that we can disregard the effects of bubbles on rheological measurements. The quenched melt filled crucibles were embedded

in epoxy, cut in half along the axis of the cups, and polished for inspection on the EPMA. Backscattered images of the different samples were then investigated for crystal-content using the software ImageJ.

To confirm the crystallinities of melts across the experimental temperature range we also collected a series of representative photomicrographs on samples from isothermal dwell experiments at those respective 1-atm temperature conditions (see Fig. 6). The lowest viscosities—on the order of 10 Pa s and lower—are determined at a temperature of 1250 ˚C. The viscosity of the melt increases with falling temperature to the point (at 1075 ˚C) that it "locked" up the rheometer when a burst of crystallization occurred in the system. Independent heating stage experiments at this temperature show that indeed the melt crystallizes profusely, thereby providing an explanation of when viscosity heightens and becomes subject to the stress imparted on the fluid by crystals.

A final pull experiment was performed at a temperature of about 1275˚C to establish the potential onset of crystallization in rheology experiments. This involved equilibrating the melt in the Pt crucible at 1275˚C for about 1 hour, inserting the spindle and allowing it to thermally adjust, and finally raising the spindle upward and out of the furnace where it was plunged into a cold water bath. This produced a pure glass that upon inspection with the EPMA revealed only sparse oxide microlites. We therefore conclude that the onset of crystallization in this basanite at 1-atm conditions occurs at T<1275˚C.

Geochemical and Analytical Techniques

We measured bulk and glass geochemical compositions using a range of techniques. Natural samples represent fresh, unaltered eruption products that were no older than a few hours upon collection and sequestration in sample containers. Thus we can disregard the effects of environmental waters on the analyses.

All analyses were conducted at the Department of Geoscience of the University of Mainz. Bulk rock analyses of tephra were conducted by XRF analysis (Table 1) while *in situ c*hemical analyses of natural samples and products of the crystallinity experiments were carried out using a JEOL JXA 8200 electron microprobe. EPMA analysis protocols of both, glass and silicate phase analyses included an acceleration voltage of 15 kV and a beam current of 12 nA. Glasses were analyzed for all major elements and additional F, Cl and SO_3 using a beam diameter of 10 µm and 5 µm for glass inclusions. Element specific dwell times were: Si 25s, Al 40s, Na 20s, K 30s, Ca 30s, Fe 60s, Mg 30s, Mn 50s, Ti, 30s, F 120s, Cl 30s, P 40s, and S 40s. Glass analyses were calibrated using the following standards: VG-A99 (Si, Fe), VG-2 (Ca, Mg), MnTi (Mn, Ti), orthoclase (Al, K), tugtupite (Na, Cl), SrF_2 (F), ZnS (S), and apatite (P). Survey measurements on glass were carried out to assess Na-drift but revealed no significant effect of Na-loss.

Silicate phases were analysed using a beam diameter of 5 µm and dwell times were 20s for each element. Calibration reference materials used with the silicate program were: wollastonite (Ca, Si), albite (Na), orthoclase (K), and MnTiO3 (Mn, Ti). Al, Cr, Fe and Mg were calibrated using the respective oxide phases. Reference materials VG-2, VG-A99 and a natural obsidian standard were analyzed repeatedly during each session analysing glass, while diopside, wollastonite and orthoclase standards were used for silicate phase measurements. Matrix correction was carried out for glass and silicate phase analyses using the ZAF and PRZ methods, respectively.

A compilation of the analytical results are reported in Table 1. All clinopyroxenes are of diopside composition and show only small differences between rim, core and, microlite compositions (Fig. 4). Plagioclase compositions are relatively homogeneous with X_{An} 0.58 – 0.65 in Tephra 1-3 and X_{An} 0.64 – 0.70 in Tephra 4. Olivine compositions are also relatively homogeneous with Mg# 0.79 – 0.83 in all natural samples.

Experimental basanite glasses obtained by rapidly quenching samples of melts during rheological assessments were analyzed for their hydrous component concentrations by way of Fourier Transform Infrared Spectroscopy (FTIR). We performed these analyses on doubly polished glass wafers prepared from coarse glass fragments that were quenched from three different viscosity experiments. Glasses were for the most part crystal-free, however one of the glass samples contained appreciable Fe-Ti oxides. Spectra from this sample were noisy and eventually discarded. The glass wafer thicknesses from the two remaining experiment glasses, measured with a Mitutoyo micrometer, were 186 and 350 µm, and each wafer yielded clean spectra in the mid-IR region. All measurements were made on a Thermo-Nicolet FTIR bench with an attached Continuum microscope that housed a liquid-Nitrogen-cooled MCT detector. The appropriate spectral range to resolve the 3565 cm^{-1} peak, corresponding to the fundamental OH-group and molecular H_2O stretching modes, was achieved through the use of a KBr beamsplitter. Transmission measurements were made with 256 scans, and using a free-path background spectrum for normalizing unknown spectra. Background spectra were collected every 30 minutes. All spectra were collected at 4 cm^{-1} resolution and resultant peak heights were measured directly on spectra within the Omnic Software interface. Peak heights, corresponding to absorbance at the 3565 cm^{-1} peak, were generally subtle, ranging from just 0.029 to 0.081 depending on the sample. These peak absorbance values were nonetheless useful for yielding quantitative water contents from about 0.005 to 0.02 wt.%, values that were calculated from the Beer's law relation and utilizing extinction coefficients for basanite glass published in ref. 3. We assumed a glass density of 2700 g cm^{-3} for all Beer's Law calculations.

Geothermobarometry and hygrometry

The glass compositions of each sample was averaged from the microprobe analyses and employed with individual clinopyroxene analyses by applying equation 33 of ref. 4 in concert with the geobarometer of ref. 5. For the calculation of eruption and rheological conditions (i.e., to narrow down a window of relevant temperatures to which viscosity measurements may be applicable), analyses of clinopyroxene rims and matrix microlites were used, interpreted of being representative of the most recent melt-crystal equilibrium. The glass compositions measured by EPMA were mostly anhydrous (all totals >99.2 wt%, most >100 wt%) and, together with the cpx compositions, yield anhydrous crystallization conditions of ~1161 ± 9 °C / 9.5 ± 0.6 kbar (n=8) for Tephra 1-3 and ~1169 ± 26 °C / 6.9 ± 2.4 kbar (n=7) for Tephra 4 (Table 1). However, considering the eruption behaviour (e.g., explosive activity and lava fountaining) the magma must have comprised at least some H_2O. Therefore, in order to obtain an estimate of magmatic water content during the eruption of Cumbre Vieja Basanite, we employed the improved calibration of the plagioclase-liquid hygrometer of ref. 6. Average compositions of matrix plagioclase crystals used with the hygrometer are X_{An} 0.63 (Tepha 1-3) and X_{An} 0.68 (Tephra 4). Both, the clinopyroxene-liquid geothermobarometer and the plagioclase-liquid were then applied iteratively to determine the magmatic water content and the hydrous cpx crystallization conditions. The calculated water content arrived at ~0.8 wt.% for both samples, at which the cpx crystallization temperature decreased slightly to 1149 ± 9 °C / 9.4 ± 0.6 kbar for Tephra 1-3 and 1157 ± 25 °C / 6.8 ± 2.3 kbar for Tephra 4.

These conditions are considered to represent the range of storage pressures and the temperatures during ascent and eruption and are broadly consistent with former studies of the La Palma volcano[7-9]. Analyses of clinopyroxene cores of Tephra 1-3 were additionally investigated and employed together with glass inclusion data of primitive composition of the same samples (Table 1) to calculate a possible onset temperature of crystallization. These conditions were determined to be ~1200 ± 23 °C / 9.4 ± 2.5 kbar for anhydrous and ~1187 ± 22 °C / 9.2 ± 2.5 kbar (n=9) for hydrous (~0.8 wt.%) melt.

Equilibrium between all the investigated cpx – liq composition pairs was ensured by examination of both, the Fe-Mg exchange between melt and crystals and the DiHd components (Diopside-Hedenbergite)[4]. Only cpx – liq composition pairs that were within 10 % of DiHd component equilibrium and at the same time satisfying that the measured $K_D(\text{Fe-Mg})^{cpx\text{-}liq}$ value was within ± 25% of the predicted temperature-corrected $K_D(\text{Fe-Mg})^{cpx\text{-}liq}$ (eq. 35, ref. 4) were included in the geothermobarometry and hygrometry calculations.

In addition to clinopyroxene–liquid geothermobarometry described above, olivine (ol)–liquid (liq) pairs were investigated to determine magma temperature. The results are however equivocal. We applied several published olivine – liquid thermometers to our dataset including models using solely the melt (glass) composition for the calculations[10-12], and those using both, liquid and olivine

compositions (ref. 4 eq. 22; ref. 13). The results indicate a broad range of temperatures: from around ~1100 °C up to ~1185 °C, dependent on the sample, and conditions such as pressure (7 – 10 kbar), and H_2O content (0 – 0.8 wt.%). Results are also highly dependent on which model was applied. We considered the models of ref. 13 and equation 22 of ref. 4 to be best suited for anhydrous and hydrous melts, respectively, as these are widely used in the published literature, and include a large number of olivine – liquid compositions in the calibrations. These models yield anhydrous temperatures of 1160 – 1184 °C, and 1100 – 1144 °C for hydrous conditions, being roughly in line with temperatures estimated by cpx – liq thermometry (ie., those described in the section above).

However, equilibrium tests based on the comparison of predicted vs. measured $K_D(Fe\text{-}Mg)^{ol\text{-}liq}$ partition coefficients[4] indicate that the olivine crystals of the studied samples are not equilibrated with the coexisting melt. Disequilibrium is further evidenced by slight zoning and weak resorption of olivine crystals in the studied samples (Fig. 5c main manuscript). Therefore, we do not to use the calculated ol-glass temperature estimates in assessments of permissible melt viscosity based on rheometry experiments. However, these data (and their shortcomings related to disequilibrium) are discussed further in the methods section of the manuscript.

Textural measurements to determine crystal size and shape distributions of natural tephra and application of an effective viscosity rheological model.

We have performed textural measurements on six tephra clasts to quantify the crystallinities, size distributions, and crystal shapes, in order to determine quantitative textural inputs, including the maximum crystal packing fraction and polydispersity, to the rheological model developed by ref. 2. The tephra clasts represent the range of crystallinities and mineralogies observed in the sample set. The model[2] accounts for all aspects of crystals' effect on viscosity, and is an elaboration on the suspension rheology model of ref. 14 with the advantage that it calculates physical parameters (e.g., maximum packing fraction and polydispersity) from textural measurements made on routinely gathered backscattered electron images (see Supplementary Figure 2, frame A, below).

Textural quantification was performed using ImageJ software and by using the following protocol. Starting with BSE photomicrographs obtained with the EPMA at a total magnification of 100X, we first filled all vesicles with black paint and then thresholded the image to isolate or segment the vesicles from groundmass glass and crystals (Frames A-C, Supplementary Figure 2). ImageJ was then used to measure the area of these vesicles. The image was then inverted and the same measurement was performed on the groundmass (Frame D, Supplementary Figure 2). The total area of vesicles minus the area of the groundmass is the corrected reference area upon which all subsequent textural parameters are derived (e.g., crystallinity, size distribution, and shape variance). The areas, sizes, and shapes of plagioclase, Fe-Ti oxides, clinopyroxene and olivine crystals, where all determined individually by treating images with the same thresholding and segmentation routine, followed by

automatic measurement by ImageJ. An example of this protocol is depicted in the image montage in Supplementary Figure 2.

Attainment of relative viscosities involved importing textural parameters (e.g., CSD, shape distribution, and crystallinity) into a user-friendly spreadsheet published as supplementary information in ref. 2. Representative model outputs are shown in the last frame of Supplementary Figure 2.

Supplementary Figure 2. Image mosaic showing the same BSE image through different stages of image analysis to determine the crystal size and shape distributions of plagioclase, clinopyroxene, olivine and titanomagnetite phases. All image processing was performed using ImageJ software.

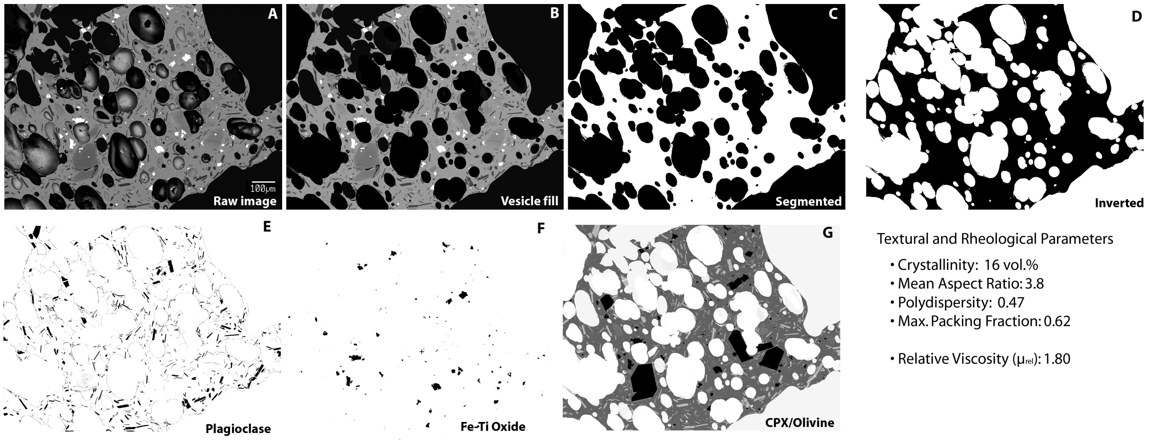

As detailed above and in the manuscript, results of these textural measurements and calculations show that the effects of the natural tephra's modest crystal contents—values that are evidenced by, and derived specifically from tephra clast textures—only shift the pure-melt viscosities to slightly higher values. The last frame in Supplementary Figure 2 shows representative outputs of the model of ref. 2, including a relative viscosity value for this sample of about 1.8. The relative viscosities (ie., the suspension viscosity normalized by the pure-melt viscosity) for all six samples range from 1.3 to 1.9. These values represent shift factors that amount to subtle increases in effective viscosity over the pure-melt values.

Videography and lava flow regime assessment

We used an open access video from the RTVC Spanish/Canary Island media collective as the basis for flow velocity and channel dimension estimates. The video was part of a live streaming feed that was filmed on 18 November 2021 at approximately 20:00-20:30 and on 25 November 2021 at about 09:26 hours. Details of the cameras' optical characteristics are unknown, however, the activity was filmed from a distance of approximately 5 km southwest of the vent in the town of Tajuya. In addition, field photos and other digital videos were taken by the authors

of this work from more northerly vantage points (in the city of El Paso) which are oriented approximately perpendicular to the direction of the RTVC camera, thereby providing a second dimension such that the approximate scale of the lava flow path and slope could be assessed. The main RTVC video of the central pyroclastic cone (Fig. 2) depicts two newly formed vents on the west side and downslope from the main long-lived vents. The two bubbling vents fed two independent yet parallel flowing lava streams that descended a steep rampart for about 100 m. We estimated the length of the cascade slope using the oblique views of the activity. The hydraulic phenomena were observed within each channel and appeared to be linked to velocity increases (accelerations) that the flows experienced as they progressed away from the sources. The features of note are the arcuate structures that periodically form within close proximity (<30 m) to the lava source. The perimeters of these arcuate structures became brighter as they moved downslope, while the interiors remained relatively darker (Fig. 2). These bright perimeters are interpreted as blunt flow fronts that reflect hydraulic jumps created as a turbulent dissipation of the flows' downslope momentum. These structures served as a useful indication of the position and progress of the flow with time and thus were used to estimate flow velocity. Because our view of the video is not perfectly orthogonal to the sloping cascade, and some uncertainty to the true length of the lava run stemming from measurement errors for slope, the velocity estimates will be apparent velocities and *less* than the true velocities. The apex of the arcuate structure (resembling ogives on lava flows) was tracked frame-by-frame during the video analysis. The travel times were documented with a stop-watch, initiated at the first signs of brightness on the leading edge of the structures. The effective distance is less than the ~100 m fall distance because the structures typically developed after a short run out distance from the source (Fig. 2). We chose an average run distance of about 70 m to account for this behavior.

Lava thickness determinations are important for calculating parameters for Reynolds number estimates (e.g., hydraulic diameter and wetted perimeter). For this purpose we sought evidence for lava flow thicknesses in online video of the 25 November 2021 lava outbreak, which occurred directly south of the main eruption center. As we do not have copyrights for these videos, nor can we confirm who the owners of the videos are, we direct the reader to the link listed below, which shows various views of the activity on 25 November 2021. Note particularly the view of lava at timestamps 1:21 – 1:35, which demonstrates the thinnest lava that we found in our investigations of eruption footage. We estimate that these thin lavas are on the order of 30 cm thick based on the substrate granularity, particularly that the substrate comprises ash to lapilli-sized particles that the lavas are flowing over, in addition to the scale of nearby sage-like shrubs, which we estimated in the field near El Paso to be on the order of 0.4 – 0.5 m tall. These lava thickness estimates provide a minimum possible thickness for the lavas observed in the cascades (erupted on 18 November 2021; Fig. 2).

Link: https://www.youtube.com/watch?v=R7Y9sFuXdYQ

A screen shot of the thin lava is provided below

Supplementary Figure 3. A screen grab image of a video collected of the Cumbre Vieja lava on 25 November 2021. This view and others like it were used to assess approximate minimum flow thickness that would then be used to inform Reynolds number calculations. Based in part on the lava observed below, we chose a minimum thickness of 30 cm for the *Re* calculations outlined in the manuscript for lavas observed in the cascades (Fig. 2 main manuscript). The cascading lava flows also appeared to be thin and wide, resembling lava sheets flowing across a relatively flat substrate. In the image below, one can recognize small shrubs of ~0.4- 0.5 m height, and pyroclasts (lapilli) in the substrate to serve as approximate scales. The lavas in the background appear to be on the order of ~0.5 – 1.0 m thick.

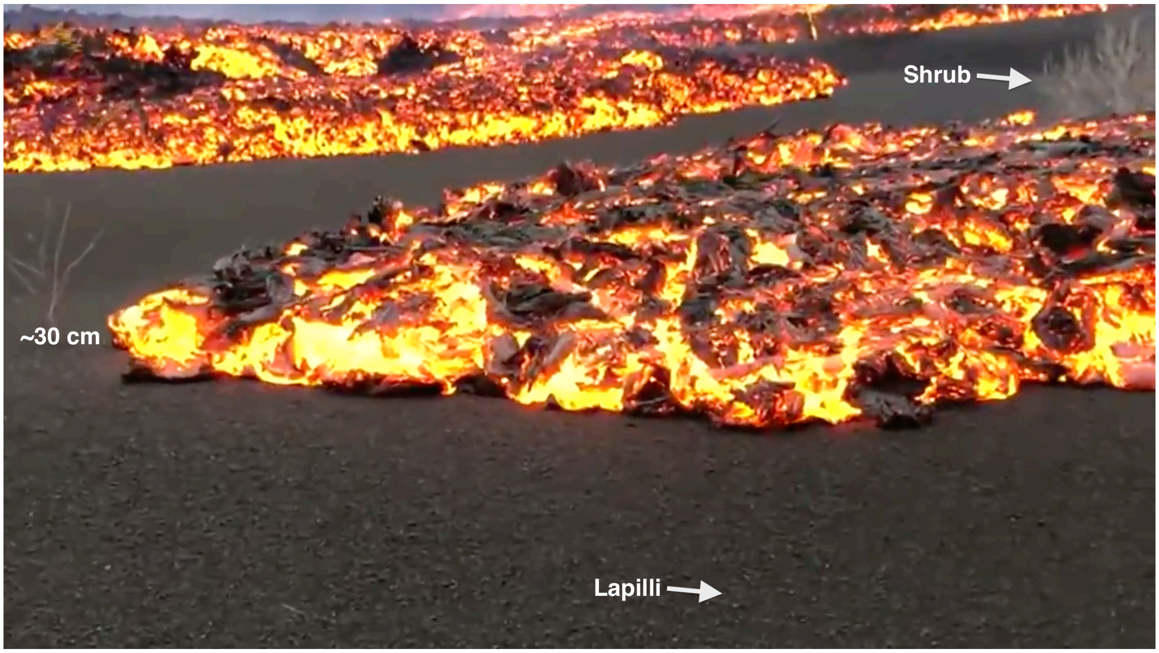

Froude and Reynolds number calculations

With the data from the dynamic video analysis in hand, we aimed to establish two characteristics about the flow regimes of emergent basanite magma. Firstly, we wanted to know the approximate Reynolds number (Re) such that the relative importance of inertial versus viscous forces could be assessed; by extension this determination permits describing the flow as laminar (Re<2000) or turbulent (Re>2000)[15]. Secondly, we wanted to establish the relative importance of inertial forces of fluid elements to the weight of the flow. This relation is described by the Froude number (Fr) and permits the delineation of a flow into critical (Fr=1), sub-critical (Fr<1) and super-critical (Fr>1) regimes. Under super-critical flow conditions, the lava may exhibit standing waves, points of super-elevation, and hydraulic jumps[1]. Ranges of these Re and Fr values were determined using the experimentally established viscosities, density based on major element

composition[16], and velocities derived from video analysis. In addition, the characteristic dimensions relating to approximate lava structural features and channel widths account for natural variability[17].

Supplementary Figure 4. Image montage from RTVC video footage of the standing-wave hosted lava flow erupted on 25 November 2021. This view is the same as depicted in Fig. 3 of the manuscript with standing waves visible on the left hand side of the main flow channel. All time stamps are in the format of: minutes:seconds.fractional seconds. Thus this montage shows a very short time period over which a lava ball (encircled and indicated with arrows) travels an approximate apparent distance of 8.4 meters. This distance was partially assessed from the size of the lava ball, which appears to be on the order of ~0.5 m in diameter based on comparisons of its size with the thin lateral flows indicated in the first frame. Although we have no direct measurements of flow thicknesses, the aspect ratio (thin and wide) along with other field evidence suggests these are rather thin (~0.5 m) overflow sheets from the main or nearby channels. The salient details of video analysis leading to a flow velocity determination are shown in the last frame on the right, and suggest that lava in this channel had at times flowed with velocities of at least 7 m sec^{-1}.

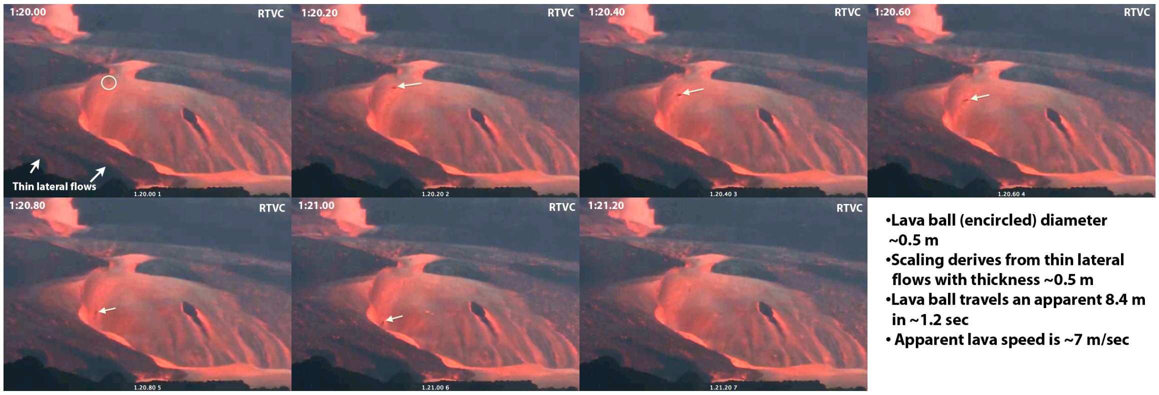

References

1. Griffiths, R.W. The dynamics of lava flows. *Annu. Rev. of Fluid Mech*., **32**, 477-518 (2000).

2. Klein, J., Meuller, S.P., Helo, C., Schweitzer, S., Gurioli, L., Castro, J.M. An expanded model and application of the combined effect of crystal-size distribution and crystal shape on the relative viscosity of magmas. *J. Volcanol. Geotherm. Res.,* **357**, 128-133 (2018).

3. von Aulock, F.W., Kennedy, B.M., Schipper, C.I., Castro, J.M., Martin, D.E., Oze, C., Watkins, J.M., Wallace, P.J., Puskar, L., Bégué, F., Nichols, A.R.L., Tuffen, H. Advances in Fourier transform infrared spectroscopy of natural glasses: From sample preparation to data analysis. *Lithos,* **206-207**, 52-64 (2014).

4. Putirka, K.D. Thermometers and barometers for volcanic systems. In: Putirka, K.D., Tepley, F. (Eds), Minerals, Inclusions, and Volcanic Processes: *Reviews in Mineralogy and Geochemistry,* **69**, 61-120 (2008).

5. Neave, D.A., Putirka, K.D. A new clinopyroxene-liquid barometer, and implications for magma storage pressures under Icelandic rift zones. *Am. Mineral.,* **102**, 777-794 (2017).

6. Waters, L.E., Lange, R.A. An updated calibration of the plagioclase-liquid hygrometer-thermometer applicable to basalts through rhyolites. *Am. Mineral.,* **100** (10), 2172-2184 (2015).

7. Weis, F.A., Skogby, H., Troll, V.R., Deegan, F.M., Dahren, B. Magmatic water contents determined through clinopyroxene: Examples from the Western Canary Islands, Spain. *Geoch., Geophys., Geosys.,* **16**, 2127-2146 (2015).

8. Klügel, A., Hoernle, K.A., Schmincke, H.-U., White, J.D.L. The chemically zoned 1949 eruption on La Palma (Canary Islands): Petrological evolution and magma supply dynamics of a rift zone eruption. *J. Geophys. Res.,* **105**, B3, 5997-6016 (2000).

9. Barker, A.K., Troll, V.R., Carracedo, J.C., Nicholls, P.A. The magma plumbing system for the 1971 Teneguìa eruption on La Palma, Canary Islands. *Cont. Mineral. Pet.,* **179**, 54 (2015).

10 Helz, R.T., Thornber, C.R. Geothermometry of Kilauea Iki lava lake, Hawaii. *Bull. Volcanol.,* **49**, 651-668 (1987).

11. Yang, H.J., Frey, F.A., Clague, D.A., Garcia, M.O. Mineral chemistry of submarine lavas from Hilo Ridge, Hawaii: implications for magmatic processes within Hawaiian rift zones. *Cont. Mineral. Pet.,* **135**, 355-372 (1996).

12. Fabbrizio, A., Spilllar, V. Methodology to derive well-calibrated thermometers: a new glass-composition geothermometer for olivine-bearing glassy samples at one atmosphere. *Ann. Geophys.,* **63**, 4, DM436 (2020).

13. Beattie, P. Olivine-melt and orthopyroxene-melt equilibria. *Cont. Mineral. Pet.*, **115**, 103-111 (1993).

14. Mueller, S., Llewellin, E.W., Mader, H.M. The rheology of suspensions of solid particles. *Proc. R. Soc. A: Math. Phys. Eng. Sci.*, **466,** 1201-1228 (2010).

15. Chow, V.T. Open-channel Hydraulics. McGraw-Hill, New York doi: ISBN 07-010776-9 (1959).

16. Lange, R.A., Carmichael, I.S.E. Thermodynamic properties of silicate liquids with emphasis on density, thermal expansion and compressibility. In: Russell J.K., Nicholls, J. (eds) Modern methods of igneous petrology: understanding magmatic processes. *Reviews in Mineralogy*, **24**, 25-64 (1990).

17. Le Moigne, Y., Zurek, J.M., Williams-Jones, G., Lev, E., Calahorrano-Di Patre, A., Anzieta, J. Standing waves in high speed lava channels: A tool for constraining lava dynamics and eruptive parameters. *J. Volcanol. Geotherm. Res.*, **401**, 106944 (2020).
