# Supplementary material for: Eruption of ultralow-viscosity basanite magma at Cumbre Vieja, La Palma, Canary Islands
Source: Nat Commun. 2022 Jun 8;13:3174. doi: 10.1038/s41467-022-30905-4 (PMC9177865; doi:10.1038/s41467-022-30905-4)
Supplement: Supplementary file 3 — Description of Additional Supplementary Files [file 41467_2022_30905_MOESM3_ESM.pdf]

Description of Additional Supplementary files

File name: Supplementary movie 1

Description: Dual cascading lava streams erupted on 18 November 2021

File name: Supplementary movie 2

Description: Lava flows erupted on 25 November 2021 exhibiting standing waves
